# Case Report: Mature cystic teratoma of the diaphragm in an adult male: a rare case and literature review

**DOI:** 10.3389/fonc.2026.1847448

**Published:** 2026-05-29

**Authors:** Xueming Zhou, Xue Li, Lusheng Wei, Shixiong Hu

**Affiliations:** 1 Department of Comprehensive Surgery, Guangdong Provincial People’s Hospital, Guangdong Academy of Medical Sciences, Southern Medical University, Guangzhou, China; 2 School of Medicine, South China University of Technology, Guangzhou, China

**Keywords:** case report, diaphragm, pathology, surgical management, teratoma

## Abstract

**Background:**

Teratomas are germ cell tumors that most commonly arise in the ovaries and testes, whereas the diaphragm is an exceptionally uncommon site of origin. To date, very few cases of diaphragmatic teratomas have been reported worldwide. The rarity and non-specific clinical presentation of these tumors often pose significant diagnostic and reconstructive challenges.

**Case presentation:**

A 34-year-old man presented with mild subxiphoid traction discomfort. Laboratory tests revealed an isolated elevation of serum carbohydrate antigen 19-9 (CA19-9) at 401.00 U/mL. Contrast-enhanced computed tomography demonstrated a 5.1 × 4.5 cm mixed cystic-solid mass on the left hemidiaphragm. The patient successfully underwent laparoscopic complete excision of the mass. Because the extensive dissection inherently destroyed the left crus and enlarged the esophageal hiatus, a primary crural repair with concurrent prophylactic Nissen fundoplication was performed to prevent iatrogenic hernia and refractory reflux. Histopathological examination confirmed a mature cystic teratoma of the diaphragm. At the 3-month follow-up, the patient remained completely asymptomatic, without dysphagia or reflux, and demonstrated normalized CA19–9 levels with no radiologic evidence of recurrence.

**Conclusions:**

Isolated CA19–9 elevation may be associated with rare primary diaphragmatic teratomas. Furthermore, concurrent laparoscopic hiatal reconstruction (e.g., Nissen fundoplication) provides an excellent, structurally sound functional cure for large tumors intimately adhering to the diaphragmatic crus.

## 1 Introduction

Teratomas typically arise in the gonads, mediastinum, or retroperitoneum (1–3). Primary teratomas originating within the diaphragm are exceedingly rare (4). Because of their low incidence and nonspecific clinical manifestations, these tumors are often detected incidentally on imaging or misdiagnosed as diaphragmatic cysts, neurogenic tumors, or paraesophageal lesions (5).

Here, we report a rare case of a primary mature cystic teratoma of the diaphragm in a 34-year-old man, successfully treated with laparoscopic resection and concurrent Nissen fundoplication, to improve clinical recognition of this rare entity.

## 2 Case description

### 2.1 Patient information and clinical findings

A 34-year-old man presented to Guangdong Provincial People’s Hospital with a 12-day history of mild subxiphoid traction discomfort. Physical examination was unremarkable; specifically, abdominal examination revealed a soft, non-tender abdomen with no palpable masses or organomegaly, and bowel sounds were normal. Chest auscultation demonstrated clear, bilateral breath sounds. Routine organ function tests were within normal limits, and the patient denied any cardiopulmonary or gastrointestinal symptoms.

### 2.2 Diagnostic assessment

**Figure 1:**
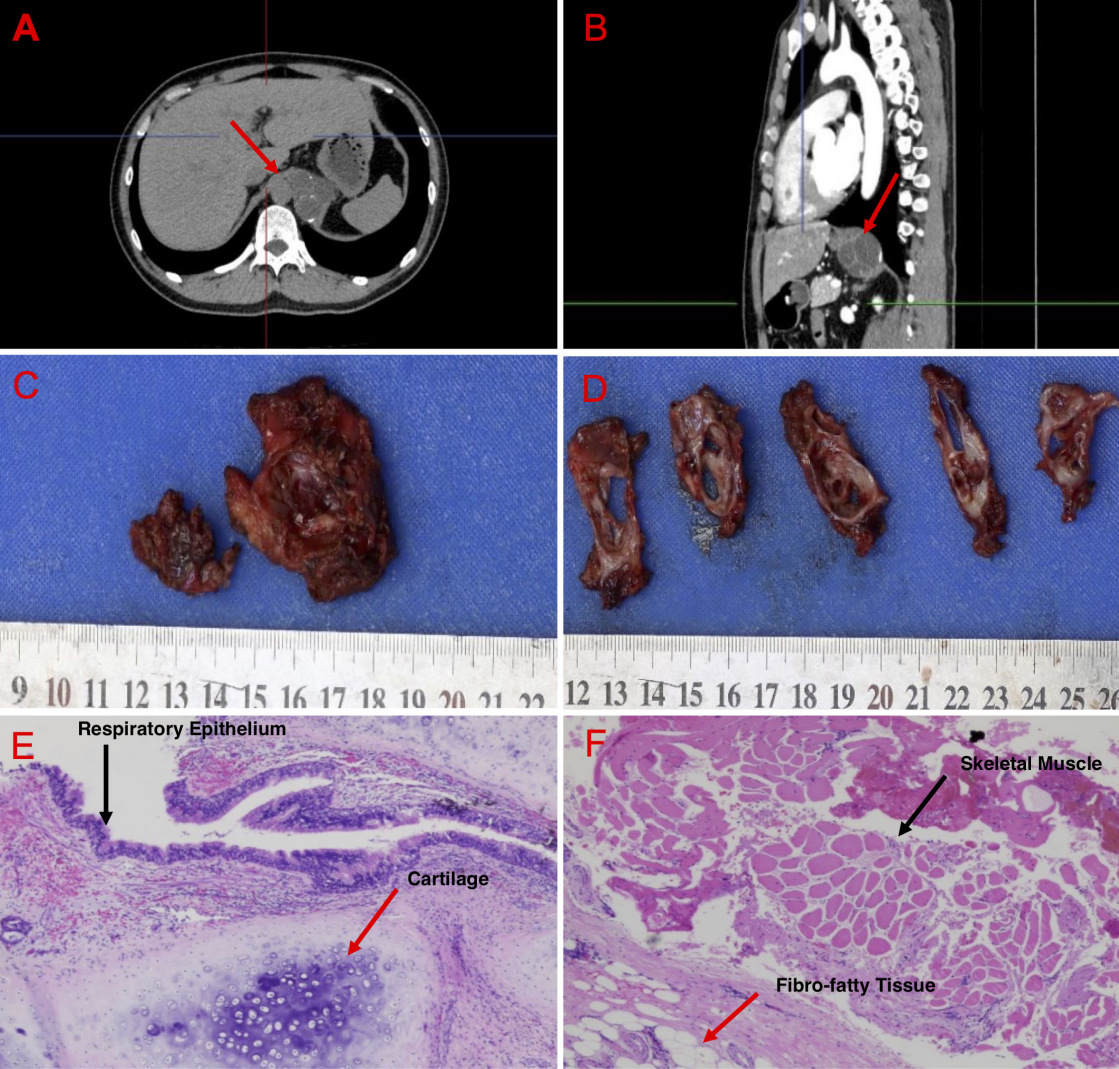
Preoperative imaging, macroscopic appearance, and histopathological examination of the primary diaphragmatic mature cystic teratoma. **(A, B)** Contrast-enhanced chest computed tomography (CT) in axial **(A)** and sagittal **(B)** views demonstrating a mixed cystic-solid mass (red arrows) located at the left hemidiaphragm. **(C, D)** Macroscopic appearance of the resected specimen. The intact mass **(C)** measures approximately 5 cm in its greatest dimension. Serial sectioning **(D)** reveals a multiseptated cystic and solid internal structure. **(E, F)** Histopathological examination (Hematoxylin and Eosin stain). **(E)** The cyst wall is lined by respiratory-type epithelium (black arrow), with underlying mature hyaline cartilage (red arrow). **(F)** The solid component contains abundant mature skeletal muscle bundles (black arrow) interspersed with mature fibro-fatty tissue (red arrow). No immature elements or malignant cells were identified.

Tumor marker profiling revealed an isolated elevation of carbohydrate antigen 19-9 (CA19-9) at 401.00 U/mL (reference range: ≤30 U/mL), while alpha-fetoprotein (AFP), carcinoembryonic antigen (CEA), and carbohydrate antigen 72-4 (CA72-4) levels remained within normal limits. Comprehensive biochemical panels and full abdominal CT scans revealed no abnormalities in the hepatobiliary or pancreatic systems, effectively ruling out common causes of CA19–9 elevation in these organs. Contrast-enhanced chest CT demonstrated focal thickening of the left hemidiaphragm with a 5.1 × 4.5 cm mixed cystic–solid mass. The lesion exhibited heterogeneous density, internal calcifications, septations, and mild heterogeneous enhancement (**Figures 1A, B**). Subsequent whole-body PET-CT showed mildly increased fluorodeoxyglucose (FDG) uptake within the mass, with no other hypermetabolic lesions detected. The rationale for performing PET-CT was to further evaluate the metabolic activity of the mass and exclude potential occult primary malignancies or systemic metastases. A significant diagnostic challenge in this case was the preoperative differentiation of the diaphragmatic mass from other more common lesions, such as bronchogenic cysts, neurogenic tumors, or paraesophageal lesions. Our diagnostic reasoning integrated the mixed cystic-solid CT features, the presence of internal calcifications, and the isolated CA19–9 elevation, which collectively pointed toward a primary diaphragmatic neoplasm (likely a teratoma), although a definitive diagnosis remained elusive until histopathological confirmation. These findings strongly suggested a primary diaphragmatic neoplasm, and surgical resection was indicated.

### 2.3 Therapeutic intervention

Laparoscopic exploration identified a 5-cm cystic mass at the left diaphragmatic crus. After liver retraction, the gastroesophageal mesentery was dissected along the lesser curvature, preserving the hepatic branch of the vagus nerve. Both diaphragmatic crura and the phrenoesophageal ligament were dissected to optimally expose the mass, which was then completely excised along its capsule (**Figures 1C, D**). Because extensive dissection around the esophageal hiatus risked iatrogenic hernia and reflux, a primary crural repair using 2–0 non-absorbable barbed sutures, augmented by a concurrent prophylactic Nissen fundoplication utilizing 2–0 non-absorbable Prolene sutures, was performed to preserve postoperative anatomic stability.

### 2.4 Pathological findings and follow-up

The histopathological examination identified well-differentiated tissues derived from multiple germ layers, including respiratory-type epithelium (endoderm), cartilage and bone (mesoderm), and fibro-fatty tissue. Crucially, extensive sampling confirmed the complete absence of immature elements or malignant cells, establishing the diagnosis of a primary mature cystic teratoma of the diaphragm (**Figures 1E, F**).

**Figure 2:**
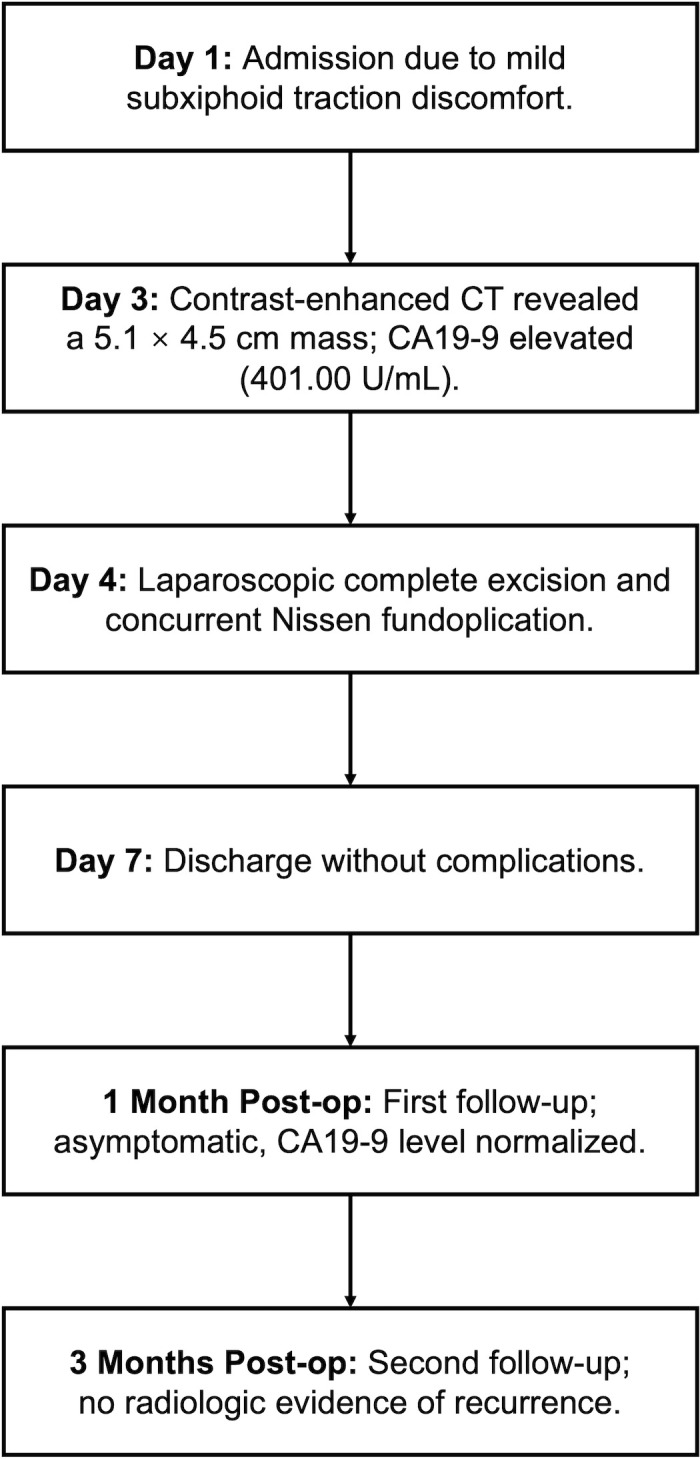
Overview of the patient’s clinical timeline. This flowchart delineates the chronological sequence of events, including initial admission, diagnostic evaluation, surgical intervention, and postoperative follow-up.

At the 1-month and 3-month follow-ups, he remained asymptomatic, with no clinical or radiologic evidence of recurrence. The CA19–9 level had normalized. The patient demonstrated excellent adherence to the follow-up schedule and tolerated the surgical intervention and recovery process well, with no adverse or unanticipated events reported during the entire postoperative period. Long-term surveillance is ongoing. The sequential clinical course, from initial presentation to the 3-month follow-up, is summarized in the timeline (**Figure 2**).

### 2.5 Patient perspective

The patient expressed satisfaction with the surgical outcome, noting the complete resolution of his subxiphoid discomfort and the absence of new gastrointestinal symptoms post-surgery.

## 3 Discussion

Mature teratomas are composed of well-differentiated tissues derived from one or more embryonic germ layers. While typical extragonadal sites include the mediastinum, sacrococcygeal region, and retroperitoneum, primary diaphragmatic involvement in adults is exceptionally rare (6). A PubMed search identified very few reported cases of primary diaphragmatic teratomas in the literature (**Table 1**) (7–9). The extreme rarity of these tumors poses diagnostic challenges and limits understanding of their biological behavior.

**Table 1:** Summary of published cases of primary diaphragmatic mature cystic teratomas in the English-language literature.

Author, year	Age	Sex	Clinical presentation	location	Treatment	Cystic teratoma size
Müller, 1986	65	F	Right upper quadrant pain and nausea	Left diaphragm	Surgical resection	8 × 12 cm
Okino H et al., 2006	45	F	Not reported	Bilateral diaphragm	Surgical resection	Left: 12 × 6.4 × 6.2 cm Right: Not reported
Ariizumi T et al., 2007	54	M	Painless mass in the left back	Left diaphragm	Surgical resection	8 × 8 cm
Current case	34	M	Mild subxiphoid traction discomfort	Left diaphragm	Surgical resection	5.1 × 4.5 cm

The pathogenesis of diaphragmatic teratomas remains speculative. The prevailing hypothesis posits that primordial germ cells migrating from the yolk sac to the genital ridge during embryogenesis may aberrantly arrest in the diaphragm (10). In our patient, comprehensive imaging confirmed the absence of a primary gonadal tumor, supporting a true primary diaphragmatic origin.

Clinically, diaphragmatic teratomas are usually asymptomatic until mass effect occurs. While classic tumor markers (AFP, CEA) are typically normal, isolated CA19–9 elevation may be associated with these tumors. This elevation likely reflects secretion from the respiratory- or gastrointestinal-type epithelial lining of the cyst (11, 12). This correlates directly with the histopathology observed in our case and explains the postoperative normalization of CA19-9. However, it is important to note that CA19–9 is not specific and can be elevated in various hepatobiliary or pancreatic conditions, which were comprehensively ruled out in our patient. Furthermore, although CA19–9 lacks specificity for teratomas, tumor marker elevation may be clinically informative, particularly in the context of potential malignant transformation, which has been reported in mature cystic teratomas (13).

Radiologically, mature teratomas present with characteristic fat, cystic components, and calcifications on CT. PET-CT generally shows mild FDG uptake, consistent with a benign etiology (14).

Surgical resection is the definitive treatment for diaphragmatic teratomas (15), enabling accurate diagnosis and preventing complications such as mass effect or the rare possibility of malignant transformation (16, 17). No recurrences have been reported following complete resection. In the present case, the tumor adhered intimately to the left diaphragmatic crus and phrenoesophageal ligament. Although resection of the diaphragmatic mass resulted in a defect approximately 5 cm in diameter, we found that direct primary crural closure (utilizing 2–0 non-absorbable barbed sutures) did not create significant tension. Given the risks of visceral erosion or infection associated with synthetic mesh, we decided against mesh reinforcement. Because the mass was located posterior to the stomach, complete excision inevitably destroyed the natural anti-reflux barrier. To prevent postoperative gastroesophageal reflux symptoms, we performed a concurrent prophylactic Nissen fundoplication (this additional procedure was explained and consented to preoperatively). This concurrent procedure effectively reconstructed the compromised anti-reflux barrier functionally. Furthermore, anatomically, the vascularized gastric wrap provided safe, autologous tissue reinforcement over the crural repair, successfully avoiding the severe risks associated with synthetic mesh. To our knowledge, such a preemptive strategy may be considered in selected cases of large perihiatal tumors.

Comparison with the three previously reported English-language cases revealed shared characteristics: tumors typically measured ≥5 cm, and nearly all occurred on the left hemidiaphragm, except for one bilateral case. These consistent features suggest potential developmental or anatomic predispositions, meriting further investigation.

Surgeons should consider diaphragmatic teratoma in the differential diagnosis of cystic lesions located near the diaphragmatic crus or esophageal hiatus, particularly when imaging reveals mixed cystic–solid features with calcifications.

This case report has certain limitations that should be acknowledged. Notably, the 3-month follow-up period is relatively short for definitively assessing long-term outcomes or potential recurrence. Furthermore, the evaluation of postoperative reflux relied on clinical symptoms rather than objective physiological assessments, such as 24-hour pH monitoring.

In conclusion, isolated CA19–9 elevation may flag rare primary diaphragmatic teratomas. Laparoscopic resection with concurrent hiatal reconstruction offers an excellent, structurally sound cure.

## Data Availability

The original contributions presented in the study are included in the article/supplementary material. Further inquiries can be directed to the corresponding authors.
